# The LANCA three-component reaction to highly substituted β-ketoenamides – versatile intermediates for the synthesis of functionalized pyridine, pyrimidine, oxazole and quinoxaline derivatives

**DOI:** 10.3762/bjoc.15.61

**Published:** 2019-03-13

**Authors:** Tilman Lechel, Roopender Kumar, Mrinal K Bera, Reinhold Zimmer, Hans-Ulrich Reissig

**Affiliations:** 1 Institut für Chemie und Biochemie, Freie Universität Berlin, Takustr. 3, D-14195 Berlin, Germany; 2 Department of Chemistry, University of Cambridge, Lensfield Road, CB2 1EW Cambridge, UK,; 3 Department of Chemistry, Indian Institute of Engineering Science and Technology, Shibpur, PO-Botanic Garden, Howrah-711 103 (WB), India

**Keywords:** allenes, condensations, multicomponent reactions, oxazoles, pyrimidines, quinoxalines

## Abstract

The LANCA three-component reaction of lithiated alkoxyallenes **LA**, nitriles **N** and carboxylic acids **CA** leads to β-ketoenamides **KE** in good to excellent yields. The scope of this reaction is very broad and almost all types of nitriles and carboxylic acids have successfully been used. The alkoxy group introduced via the allene component is also variable and hence the subsequent transformation of this substituent into a hydroxy group can be performed under different conditions. Enantiopure nitriles or carboxylic acids can also be employed leading to chiral **KE** with high enantiopurity and dinitriles or dicarboxylic acids also lead to the expected bis-β-ketoenamides. β-Ketoenamides incorporate a unique combination of functional groups and hence a manifold of subsequent reactions to highly substituted heterocyclic compounds is possible. An intramolecular aldol-type condensation reaction efficiently furnishes pyridin-4-ols **PY** that can be further modified by palladium-catalyzed reactions, e.g., to specifically substituted furopyridine derivatives. Condensations of β-ketoenamides with ammonium salts or with hydroxylamine hydrochloride afford pyrimidines **PM** or pyrimidine *N*-oxides **PO** with a highly flexible substitution pattern in good yields. The functional groups of these heterocycles also allow a variety of subsequent reactions to various pyrimidine derivatives. On the other hand, acid-labile alkoxy substituents such as a 2-(trimethylsilyl)ethoxy group are required for the conversion of β-ketoenamides into 5-acetyl-substituted oxazoles **OX**, again compounds with high potential for subsequent functional group transformations. For acid labile β-ketoenamides bearing bulky substituents the acid treatment leads to acylamido-substituted 1,2-diketones **DK** that could be converted into quinoxalines **QU**. All classes of heterocycles accessed through the key β-ketoenamides show a unique substitution pattern – not easily accomplishable by alternative methods – and therefore many subsequent reactions are possible.

## Introduction

Multicomponent reactions are known to create unique product skeletons in an atom economic, efficient and time saving fashion. In many cases, compounds bearing functional groups of relatively high energy level with the potential of multiple reactivity are employed, for instance nitriles, isonitriles or alkynes [1–9]. Not surprisingly, simple or functionalized allenes have also been used in multicomponent processes and – dependent on the substitution pattern of the allene – a remarkable variety of reactions and product types are known using the three-carbon backbone of these reactive compounds [10]. During the exploration of alkoxyallene chemistry [11–20] we accidently discovered a new three-component reaction leading to β-ketoenamides that are uniquely functionalized alkenes and suitable for a variety of subsequent reactions, in particular in heterocyclic synthesis.

**Scheme 1:**
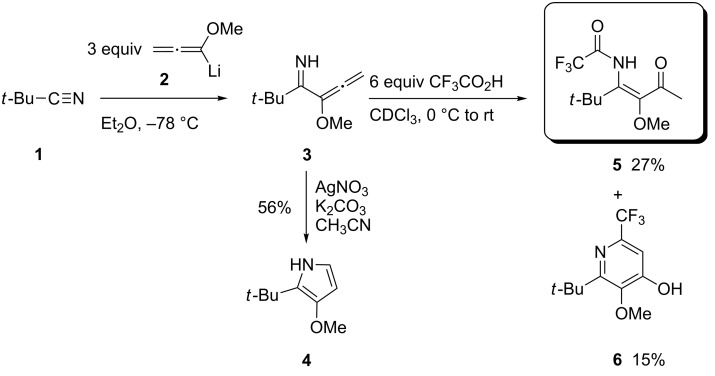
Discovery of the LANCA three-component reaction. The reaction of pivalonitrile (**1**) with lithiated methoxyallene **2** leads to allenyl-substituted imine **3** which upon reaction with trifluoroacetic acid affords β-ketoenamide **5** and pyridin-4-ol derivative **6**.

This LANCA three-component reaction (**LA** = lithiated alkoxyallene, **N** = nitrile, **CA** = carboxylic acid) was observed for the first time by Oliver Flögel, who treated pivalonitrile (**1**) with lithiated methoxyallene **2** and isolated the expected primary addition product **3** [21]. This intermediate was subjected to different cyclization conditions (Scheme 1) and the desired pyrrole derivative **4** was produced under specific conditions employing silver nitrate as catalyst. However, the treatment of **3** with an excess of trifluoroacetic acid led to a mixture of β-ketoenamide **5** and pyridin-4-ol derivative **6**. Thus, the carboxylic acid did not act as a catalyst in this reaction as expected, it was incorporated into the products!

**Scheme 2:**
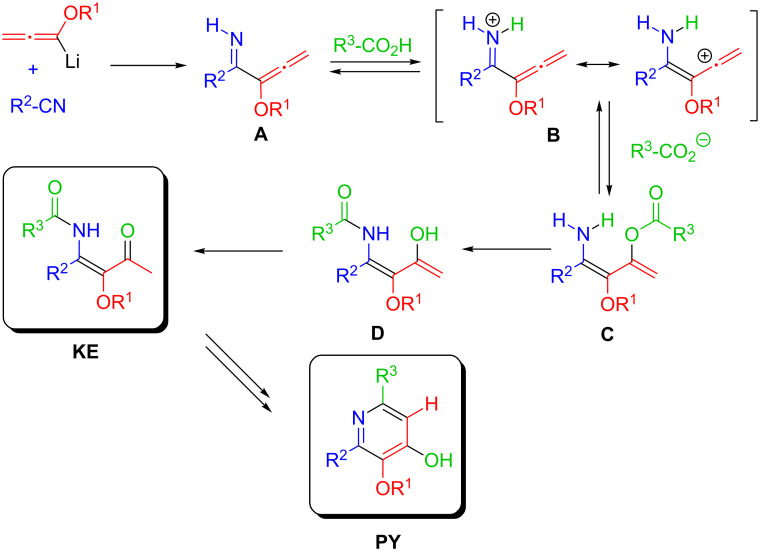
Proposed mechanism of the LANCA three-component reaction to β-ketoenamides **KE** and pyridin-4-ol derivatives **PY**.

The unique mechanism leading to the β-ketoenamide and the pyridin-4-ol has been discussed earlier [21–23], but the essentials of the involved cascade reactions have to be presented again in order to understand the formation of the crucial β-ketoenamide intermediates (Scheme 2). The protonation of the primarily formed allenylimine **A** by the added carboxylic acid at the nitrogen gives an allenyl iminium/aminobutadienyl cation intermediate **B** that accepts the present carboxylate at the electrophilic carbon to provide an acyloxy-substituted aminobutadiene derivative **C**. The acyl group is subsequently transferred to the close amino group giving **D** thus accomplishing the final connectivity of the three components. Enol/carbonyl tautomerization gives the isolated β-ketoenamide **KE** with *E*-configuration being the result of the intramolecular acyl transfer. Even after storage of β-ketoenamides there is no evidence that an isomerization to the corresponding *Z*-isomers occurs.

It should be noted here that the protonation to the allenyl iminium species **B** implies an “umpolung of reactivity” of the alkoxyallene subunit converting the central allene carbon to an electrophilic center whereas this carbon is a nucleophilic center in the neutral compound. The obtained β-ketoenamides are alkenes with a remarkable assembly of functional groups: they are enamides, enol ethers and α,β-unsaturated carbonyl compounds at the same time. In addition, their methyl ketone subunit is required for some of the subsequent transformations, e.g., the synthesis of pyridin-4-ols **PY**.

**Scheme 3:**
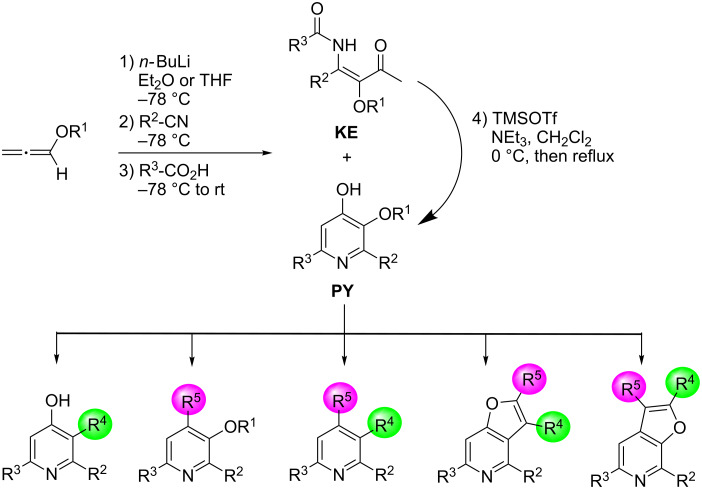
One-pot preparation of pyridin-4-ols **PY** and their subsequent transformations to highly substituted pyridine derivatives and furopyridines.

The first reaction shown in Scheme 1 gave a mixture of β-ketoenamide **5** and its subsequent cyclization product pyridin-4-ol **6** in low yields. This new route to highly substituted pyridin-4-ol derivatives could be streamlined as a one-pot procedure and after completion of the condensation reaction with trimethylsilyl trifluoromethanesulfonate (TMSOTf) and a tertiary amine as base a broad range of pyridine derivatives was accessible (Scheme 3). According to its discoverer, we named this reaction Flögel pyridine synthesis [21]. The reaction is very flexible with respect to the employed alkoxyallenes, nitriles and carboxylic acids and due to the two differently protected oxygen functions of the pyridin-4-ols these intermediates could be further substituted, e.g., through palladium-catalyzed reactions, to give highly substituted pyridine derivatives in a great variety. The scope and limitations of this approach as well as many subsequent reactions to a broad range of pyridine or furopyridine derivatives has recently been summarized in a comprehensive review [23]. It should be mentioned here, that alkoxyallenes are no exotic compounds but easily available in two steps from simple starting materials [24–25]. They can smoothly be prepared in multigram scale and recently a flow chemistry approach on the use of lithiated methoxyallene was published [26].

For the case, that substituents R^3^ are strongly electron withdrawing the relatively electrophilic amido carbonyl group of the β-ketoenamides partially undergoes a subsequent intramolecular aldol-type condensation reaction to furnish the pyridin-4-ols. Therefore, trifluoroacetic acid or related fluorinated carboxylic acids [22] lead to mixtures of the two products as shown in Scheme 1. For other carboxylic acids the multistep reaction of the three components stops at the stage of the β-ketoenamides that were usually isolated in good yields. These intermediates also provide pyridin-4-ols under slightly more rigorous condensation conditions, but they can also be used in alternative synthetic operations providing other compound classes, in particular heterocyclic compounds. The synthesis of pyrimidines **PM**, pyrimidine *N*-oxides **PO**, oxazoles **OX**, 1,2-diketones **DK** and quinoxalines **QU** starting from β-ketoenamides **KE** is the topic of the present review.

## Review

### Scope of the LANCA three-component synthesis of β-ketoenamides

**Scheme 4:**
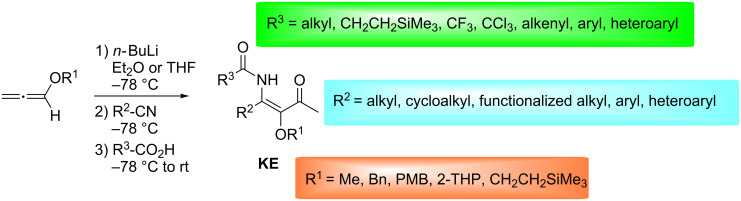
Synthesis of β-ketoenamides **KE** by the LANCA three-component reaction of alkoxyallenes, nitriles and carboxylic acids.

The scope of the LANCA three-component synthesis of β-ketoenamides **KE** through the reaction of alkoxyallenes, nitriles and carboxylic acids is very broad and only a few clear limitations were found (Scheme 4). With lithiated methoxyallene (R^1^ = Me) – the standard alkoxyallene generally used to study new reactions – many conceivable combinations of nitriles as second and carboxylic acids as third component were examined.

In Table 1 the resulting products **KE1–35** are collected, showing that simple alkyl, branched alkyl, cycloalkyl, functionalized alkyl, alkenyl, aryl or heteroaryl substituents (R^2^ or R^3^) can be introduced into the resulting β-ketoenamides **KE**. The use of α,β-unsaturated nitriles as second component did not provide reasonable results, possibly due to a competing 1,4-addition of the lithiated methoxyallene to the double bonds. On the other hand, α,β-unsaturated carboxylic acids were excellent third components as shown in various examples (Table 1, entries 15–19, 26–28, and 34). Even with acrylic acid the expected product **KE15** was isolated in 91% yield. Although we did not systematically study nitriles with heterocyclic substituents, we showed that thiophene-2-carbonitrile is an excellent substrate leading to **KE32–35** in good yields (Table 1, entries 32–35). Unfortunately, pyridine-2-carbonitrile could not be used as electrophilic component; the reason for this failure is unclear. By use of heterocyclic carboxylic acids we could smoothly introduce 2-thienyl and 2-pyridyl substituents into the β-ketoenamides **KE2**, **KE23**, **KE30**, **KE31** and **KE35** (Table 1, entries 2, 23, 30, 31, and 35).

**Table 1:** Synthesis of β-ketoenamides **KE1**–**35** through the LANCA three-component reaction of lithiated methoxyallene, nitriles (R^2^-CN) and carboxylic acids (R^3^-CO_2_H) according to Scheme 4.^a^

entry	R^1^	R^2^	R^3^	product	yield	ref.

1	Me	Me	Ph	**KE1**	13%	[27]
2	Me	Me	2-Py	**KE2**	22%	[27]
3	Me	iPr	CCl_3_	**KE3**	41% (+9% **PY**)	[28]
4	Me	iPr	Ph	**KE4**	53%	[27]
5	Me	cPr	cPr	**KE5**	75%	[29]
6	Me	cPr	C_6_H_4_-4-Br	**KE6**	53%	[30]
7	Me	*t*-Bu	allyl	**KE7**	82%	[31]
8	Me	*t*-Bu	Bn	**KE8**	95%	[31]
9	Me	*t*-Bu	cPr	**KE9**	39%	[30]
10	Me	*t*-Bu	CH_2_CH_2_SiMe_3_	**KE10**	49%	[30]
11	Me	*t*-Bu	CH_2_OPh	**KE11**	63%	[27]
12	Me	*t*-Bu	CF_3_	**KE12**	27% (+15% **PY**)	[21]
13	Me	*t*-Bu	CH_2_Cl	**KE13**	60%	[30]
14	Me	*t*-Bu	CCl_3_	**KE14**	27% (+28% **PY**)	[28]
15	Me	*t*-Bu	HC=CH_2_	**KE15**	91%	[31]
16	Me	*t*-Bu	HC=CH-Me	**KE16**	93%	[31]
17	Me	*t*-Bu	HC=CH-Ph	**KE17**	87%	[31]
18	Me	*t*-Bu	HC=CH-2-Fu	**KE18**	89%	[31]
19	Me	*t*-Bu	HC=CH-2-Th	**KE19**	80%	[31]
20	Me	*t*-Bu	C≡CH	**KE20**	72%	[27]
21	Me	*t*-Bu	Ph	**KE21**	76%	[27]
22	Me	Ad	cPr	**KE22**	67%	[32]
23	Me	CH_2_OMe	2-Py	**KE23**	33%	[27]
24	Me	Ph	CF_3_	**KE24**	30% (+28% **Py**)	[21]
25	Me	Ph	CCl_3_	**KE25**	42%	[28]
26	Me	Ph	CH=CH_2_	**KE26**	45%	[31]
27	Me	Ph	HC=CH-Ph	**KE27**	51%	[31]
28	Me	Ph	HC=CH-2-Th	**KE28**	68%	[31]
29	Me	Ph	Ph	**KE29**	45%	[29]
30	Me	Ph	2-Py	**KE30**	42%	[33]
31	Me	Ph	2-Th	**KE31**	43%	[27]
32	Me	2-Th	CH_2_OMe	**KE32**	47%	[27]
33	Me	2-Th	CH_2_Cl	**KE33**	62%	[30]
34	Me	2-Th	HC=CH-Ph	**KE34**	68%	[31]
35	Me	2-Th	2-Th	**KE35**	70%	[34]

^a^Abbreviations: Ad = 1-adamantyl, Fu = furyl, Py = pyridyl, Th = thienyl; all alkenyl substituents are *E*-configured.

The present approach does not allow the synthesis of β-ketoenamides with substituents R^2^ = H or R^3^ = H. The reaction of lithiated methoxyallene with hydrogen cyanide as second component was not examined due to the assumed Brønsted acid property of the latter. As a substitute, cyano trimethylsilane was examined, however, this experiment did not afford the corresponding β-ketoenamide. Unexpectedly, using formic acid as the third component afforded only mixtures of compounds whose structures could not be elucidated. The role of formic acid in the three-component reaction should be investigated by finding the proper substrates and conditions. The β-ketoenamides with *N*-formyl substituents should be valuable precursors for subsequent transformations.

As expected, the reactions involving trifluoroacetic acid gave only low yields of the β-ketoenamides **KE** due to the competing in situ cyclization to the corresponding pyridin-4-ols (**PY**, Table 1, entries 12 and 24). The related reactions with trichloroacetic acid provided the β-ketoenamides in slightly better yields with lower amounts of the corresponding pyridin-4-ols (Table 1, entries 3, 14, and 25) showing that the electrophilicity of the amide carbonyl group is lower in these substrates.

**Scheme 5:**
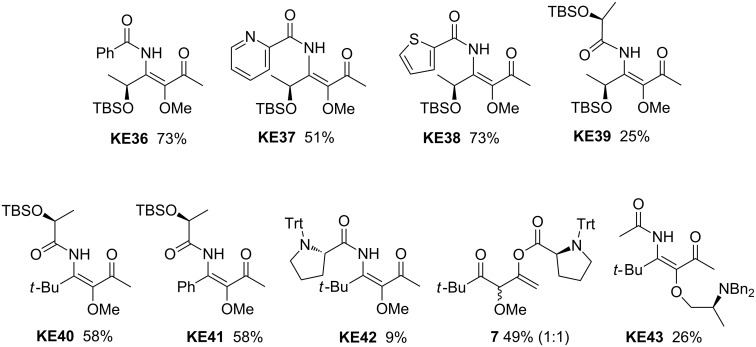
β-Ketoenamides **KE36**–**43** derived from enantiopure components.

Stereogenic centers could also successfully be introduced into the β-ketoenamides as shown by the examples collected in Scheme 5. All three possibilities to use enantiopure starting materials were examined in the three-component reaction. The products **KE36**–**39** are derived from the O-protected nitrile obtained from (*S*)-lactic acid [35–36]. This chiral acid itself was incorporated as third component resulting in β-ketoenamides **KE39**–**41**. There is no indication of an erosion of the enantiopurity and the chiral compounds were converted into the corresponding pyridin-4-ol derivatives and tested as chiral ligands in asymmetric catalysis [37]. Using the *N*-trityl-substituted proline as carboxylic acid provided the expected β-ketoenamide **KE42** in low yield and as major product we isolated compound **7** in 49% yield (1:1 mixture of the two possible diastereomers). Probably due to the bulkiness of the acyl group, the migration to the nitrogen is strongly hampered and hence the three-component cascade almost completely stops at the stage of aminobutadiene **C** (see Scheme 2) that is hydrolyzed during work-up to give α-methoxy carbonyl compound **7**. The isolation of this compound supports our mechanistic proposal as shown in Scheme 2 and it shows that sterically very hindered carboxylic acids are probably poor components in the route to β-ketoenamides. A systematic study of this possible limitation was not carried out, but a component such as Mosher acid with a tertiary carbon next to the carboxylic acid function was successfully used in the three-component reaction and the corresponding β-ketoenamide was converted into the corresponding pyridin-4-ol in good overall yield [36]. This demonstrates that carboxylic acids with tertiary centers are possible candidates for the route to β-ketoenamides.

The last example shown in Scheme 5 demonstrates that allenes with chiral alkoxy substituents are also suitable starting materials in the three-component reaction. We did not study lithiated carbohydrate-derived alkoxyallenes that were good precursors for other applications [38–39], but prepared the allene derived from N-protected alaninol that was converted into β-ketoenamide **KE43** in 26% yield [30].

It should be mentioned here that most of the reactions to β-ketoenamides were performed only once under standard conditions without detailed optimization reaction conditions such as stoichiometry of components, applied temperatures and reaction times. It is therefore very likely that in the cases where low or moderate yields were recorded improvements are easily possible. For a few examples, we also performed the reactions in larger scale, e.g., the synthesis of β-ketoenamides **KE35** that was prepared in 3.5 g quantity [34]. The scalability of the three-component reactions seems therefore no problem which is important for the multistep preparation of subsequent products (see below).

**Scheme 6:**
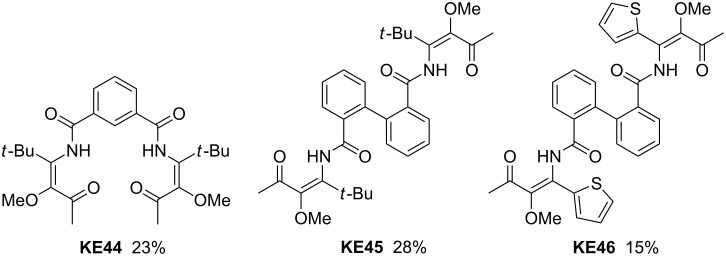
Bis-β-ketoenamides **KE44**–**46** derived from aromatic dicarboxylic acids.

Aromatic dinitriles such as 1,3- and 1,4-dicyanobenzene were also examined as second component in the three-component reaction. The obtained bis-β-ketoenamides were not isolated and purified, but directly converted into the corresponding bis-pyridin-4-ol derivatives by cyclocondensation [40]. The overall yields were only in the range of 20% probably due to solubility problems with employed aromatic dinitriles. Nevertheless, these examples showed the feasibility of this approach to highly substituted β-ketoenamides. Similar results were obtained by the use of aromatic dicarboxylic acids (Scheme 6). Again, the moderate efficacy may be due to their low solubility in ethereal solvents at low temperatures – a problem that could only partially be solved by use of DMF as cosolvent. The yields of β-ketoenamides **KE44**–**46** are only in the range of 25%, but the three-component approach to unique multifunctional products is nevertheless remarkable [41].

For subsequent reactions, alkoxy groups other than the methoxy group were desirable because the latter substituent can only be converted into free hydroxy groups by treatment with strong (Lewis) acids. We therefore examined benzyloxyallene as starting material in the three-component reaction to β-ketoenamides. The resulting products were deprotectable under milder conditions as shown below. The examples, including one with a *p*-methoxybenzyloxy group (PMB, Table 2, entry 10) are collected in Table 2. As expected there were no fundamental differences to the observations with methoxyallene. The β-ketoenamides derived from trifluoroacetic acid are only available in low yield due to the fast formation of the corresponding pyridin-4-ols (**PY**, Table 2, entries 1, 6, and 10). For the other combinations of substituents the unoptimized yields of β-ketoenamides are satisfying.

**Table 2:** Synthesis of β-ketoenamides **KE47**–**56** through the LANCA three-component reaction of lithiated benzyloxyallene, nitriles (R^2^-CN) and carboxylic acids (R^3^-CO_2_H) according to Scheme 4.^a^

entry	R^1^	R^2^	R^3^	product	yield	ref.

1	Bn	Me	CF_3_	**KE47**	5% (+39% **PY**)	[22]
2	Bn	*n*-Non	Ph	**KE48**	27%	[42]
3	Bn	cPr	cPr	**KE49**	56%	[27]
4	Bn	*t*-Bu	cPr	**KE50**	44%	[30]
5	Bn	*t*-Bu	2-Th	**KE51**	52%	[42]
6	Bn	Ph	CF_3_	**KE52**	40% (+36% **PY**)	[42]
7	Bn	Ph	Ph	**KE53**	54%	[29]
8	Bn	Ph	2-Py	**KE54**	27%	[42]
9	Bn	2-Th	2-Th	**KE55**	32%	[27]
10	PMB	Ph	CF_3_	**KE56**	23% (+10% **PY**)	[42]

^a^Abbreviations: Fu = furyl, Py = pyridyl, Th = thienyl, PMB = CH_2_C_6_H_4_-4-OMe; all alkenyl substituents are *E*-configured.

Another good alternative to methoxyallene is the 2-(trimethylsilyl)ethoxy-substituted allene. The 2-(trimethylsilyl)ethyl substituent can be removed from the products either by fluoride or acid treatment under mild conditions. Again, there were no great differences in the performance of this component compared to methoxyallene or benzyloxyallene. In Table 3 the result with tetrahydropyranyl-substituted allene is also included (Table 3, entry 1) that gave a moderate yield of β-ketoenamide **KE57**.

**Table 3:** Synthesis of β-ketoenamides **KE57**–**77** by the LANCA three-component reaction of lithiated 2-(trimethylsilyl)ethoxyallene, nitriles (R^2^-CN) and carboxylic acids (R^3^-CO_2_H) according to Scheme 4.^a^

entry	R^1^	R^2^	R^3^	product	yield	ref.

1	2-THP	cPr	cPr	**KE57**	24%	[42]
2	TMSE	cPr	cPr	**KE58**	75%	[42]
3	TMSE	cPr	CH=CH-Ph	**KE59**	57%	[30]
4	TMSE	*t*-Bu	Me	**KE60**	52%	[42]
5	TMSE	*t*-Bu	CF_3_	**KE61**	14% (+28% PY)	[33]
6	TMSE	*t*-Bu	HC=CH_2_	**KE62**	40%	[43]
7	TMSE	*t*-Bu	HC=CH-Me	**KE63**	42%	[43]
8	TMSE	*t*-Bu	HC=CH-Ph	**KE64**	35%	[43]
9	TMSE	*t*-Bu	HC=CH-C_6_H_4_-NO_2_	**KE65**	52%	[43]
10	TMSE	*t*-Bu	HC=CH-2-Fu	**KE66**	30%	[43]
11	TMSE	*t*-Bu	Ph	**KE67**	49%^b^	[30]
12	TMSE	Ad	cPr	**KE68**	57%	[42]
13	TMSE	Ph	CF_3_	**KE69**	39% (+24% PY)	[42]
14	TMSE	Ph	HC=CH-Me	**KE70**	46%	[43]
15	TMSE	Ph	HC=CH-Ph	**KE71**	50%	[43]
16	TMSE	Ph	C≡CH	**KE72**	21%	[42]
17	TMSE	Ph	Ph	**KE73**	36%	[29]
18	TMSE	Ph	2-Py	**KE74**	24%	[42]
19	TMSE	Ph	2-Th	**KE75**	75%	[42]
20	TMSE	Ph	Ac	**KE76**	28%	[42]
21	TMSE	2-Th	Ph	**KE77**	74%	[29]

^a^Abbreviations: THP = tetrahydropyranyl, Ad = 1-adamantyl, Fu = furyl, Py = pyridyl, Th = thienyl; all alkenyl substituents are *E*-configured. ^b^As second product, 15% of the imine tautomer of β-ketoenamide **KE67** was isolated.

**Scheme 7:**
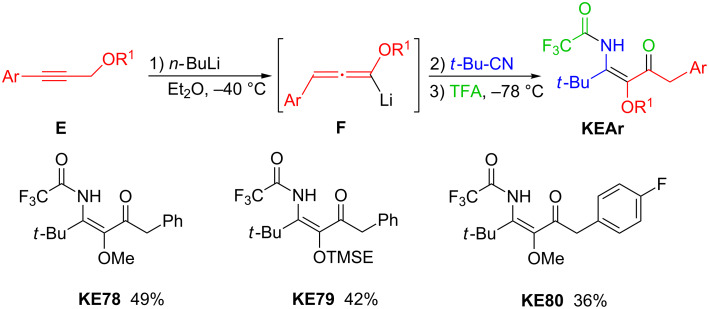
Conversion of alkyl propargyl ethers **E** into aryl-substituted β-ketoenamides **KEAr** and selected products **KE78**–**80** obtained by this route.

The alkoxyallenes so far listed are unsubstituted at the C-3 terminus and related allenes bearing alkyl groups at this carbon are not directly accessible [44]. In contrast, products that are formally derived from 3-aryl-substituted alkoxyallenes can smoothly be prepared from the corresponding alkyl propargyl ethers **E** (Scheme 7). Their deprotonation with *n*-butyllithium proceeds with a proton shift delivering the intermediate **F** that reacts with electrophiles at C-1. The three-component reaction with nitriles and carboxylic acids then leads to the corresponding β-ketoenamides **KEAr** in moderate yields. The reaction sequence is illustrated in Scheme 7 also showing the three products **KE78** [22], **KE79** [45] and **KE80** [46] that were prepared by this largely unexplored, but very promising method. It opens a route to highly substituted heterocycles as shown by the cyclocondensation of **KE78** that gave the corresponding pentasubstituted pyridin-4-ol derivative in 91% yield [22].

As mentioned above, a large number of the prepared β-ketoenamides **KE** was converted into the corresponding pyridin-4-ol derivatives **PY** and subsequent products of these versatile heterocyclic intermediates. Our published review on this topic [23] presents many examples of β-ketoenamides **KE** that were not purified but directly transferred into these pyridine derivatives. Hence, the scope of available β-ketoenamides **KE** is broader than the eighty examples presented here. This fact should be kept in mind when the reactions of β-ketoenamides to alternative subsequent products are discussed in the following chapters.

### Synthesis of pyrimidine derivatives

**Scheme 8:**
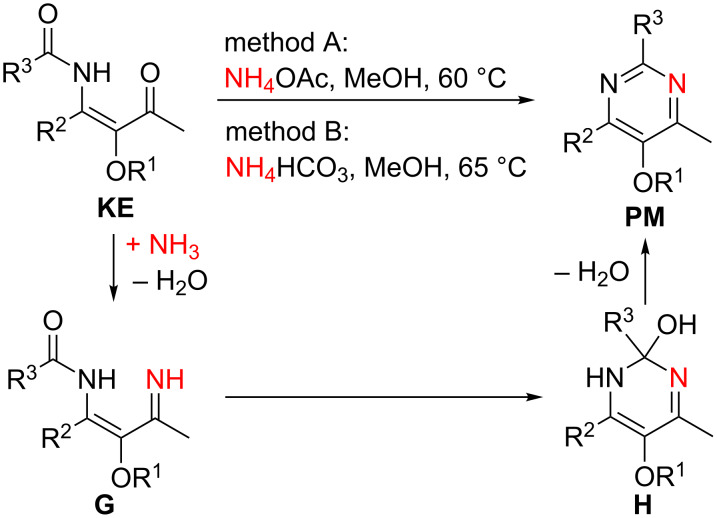
Condensation of LANCA-derived β-ketoenamides **KE** with ammonium salts to give 5-alkoxy-substituted pyrimidine derivatives **PM**.

The β-ketoenamides **KE** also serve as excellent starting materials for the preparation of highly substituted pyrimidine derivatives **PM** [47–49]. Cyclocondensation reactions with ammonium salts in methanol afford these versatile heterocycles in good to excellent yields (Scheme 8). In most cases, ammonium acetate gave the best results (method A) and in a few examples ammonium bicarbonate was tested as alternative (method B) [29,33]. The plausible mechanism of this transformation involves the formation of an α,β-unsaturated imine **G** and its cyclization to **H** followed by water elimination. As characteristic substitution pattern, the available pyrimidines **PM** contain a methyl group at C-4 and an alkoxy group OR^1^ at C-5.

The remarkably wide scope of this pyrimidine synthesis is demonstrated by the thirty examples collected in Table 4. All tested β-ketoenamides were successfully converted into the pyrimidines **PM** and the examples show that the method is fully compatible with methoxy, benzyloxy and 2-(trimethylsilyl)ethoxy substituents. The groups R^2^ and R^3^ can be unbranched, branched or functionalized alkyl, aryl or heteroaryl groups. In addition, there are many examples with alkenyl substituents R^3^.

**Table 4:** Condensation of β-ketoenamides **KE** with ammonium salts to give pyrimidine derivatives **PM1**–**30** according to Scheme 8.^a^

entry	precursor	R^1^	R^2^	R^3^	product	yield (method)^b^	ref.

1	**KE1**	Me	Me	Ph	**PM1**	40% (A)	[33]
2	**KE4**	Me	iPr	Ph	**PM2**	54% (A)	[33]
3	**KE5**	Me	cPr	cPr	**PM3**	56% (A)	[29]
4	**KE7**	Me	*t*-Bu	allyl	**PM4**	69% (A)	[31]
5	**KE8**	Me	*t*-Bu	Bn	**PM5**	82% (A)	[50]
6	**KE12**	Me	*t*-Bu	CF_3_	**PM6**	31% (A)	[33]
7	**KE15**	Me	*t*-Bu	HC=CH_2_	**PM7**	77% (A)	[31]
8	**KE16**	Me	*t*-Bu	HC=CH-Me	**PM8**	75% (A)	[31]
9	**KE17**	Me	*t*-Bu	HC=CH-Ph	**PM9**	85% (A)	[31]
10	**KE18**	Me	*t*-Bu	HC=CH-2-Fu	**PM10**	67% (A)	[31]
11	**KE19**	Me	*t*-Bu	HC=CH-2-Th	**PM11**	69% (A)	[31]
12	**KE20**	Me	*t*-Bu	C≡CH	**PM12**	55% (A)	[33]
13	**KE26**	Me	Ph	HC=CH_2_	**PM13**	55% (A)	[31]
14	**KE27**	Me	Ph	HC=CH-Ph	**PM14**	84% (A)	[31]
15	**KE29**	Me	Ph	Ph	**PM15**	73% (A), 66% (B)	[29]
16	**KE30**	Me	Ph	2-Py	**PM16**	38% (A)	[33]
17	**KE31**	Me	Ph	2-Th	**PM17**	65% (B)	[33]
18	**KE34**	Me	2-Th	HC=CH-Ph	**PM18**	78% (A)	[31]
19	**KE35**	Me	2-Th	2-Th	**PM19**	83% (A)	[50]
20	**KE53**	Bn	Ph	Ph	**PM20**	75% (B)	[29]
21	**KE55**	Bn	2-Th	2-Th	**PM21**	68% (A)	[33]
22	**KE61**	TMSE	*t*-Bu	CF_3_	**PM22**	66% (B)	[33]
23	**KE62**	TMSE	*t*-Bu	HC=CH_2_	**PM23**	53% (A)	[43]
24	**KE63**	TMSE	*t*-Bu	HC=CH-Me	**PM24**	70% (A)	[43]
25	**KE64**	TMSE	*t*-Bu	HC=CH-Ph	**PM25**	52% (A)	[43]
26	**KE66**	TMSE	*t*-Bu	HC=CH-2-Fu	**PM26**	65% (A)	[43]
27	**KE70**	TMSE	Ph	HC=CH-Me	**PM27**	66% (A)	[43]
28	**KE71**	TMSE	Ph	HC=CH-Ph	**PM28**	68% (A)	[43]
29	**KE73**	TMSE	Ph	Ph	**PM29**	86% (A)	[29]
30	**KE77**	TMSE	2-Th	Ph	**PM30**	74% (B)	[29]

^a^Abbreviations: Fu = furyl, Py = pyridyl, Th = thienyl; all alkenyl substituents are *E*-configured. ^b^Method A: NH_4_OAc, MeOH, 60 °C; method B: NH_4_HCO_3_, MeOH, 65 °C.

**Scheme 9:**
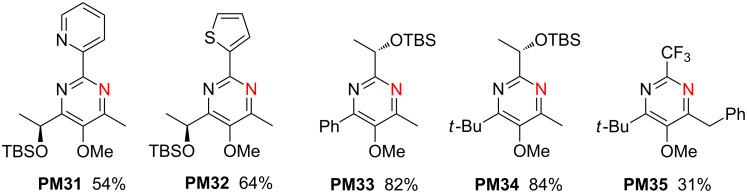
Synthesis of **PM31**–**35** from β-ketoenamides **KE37**, **KE38**, **KE40**, **KE41** and **KE78** obtained by method A (NH_4_OAc, MeOH, 60 °C).

In Scheme 9 additional examples **PM31**–**34** having stereogenic centers are presented, that were obtained from β-ketoenamides **KE37**, **KE38**, **KE40** and **KE41** (see Scheme 5) [43]. The pyrimidine **PM35** is derived from β-ketoenamide **KE78** (see Scheme 7) and bears a benzyl group at C-4 instead of the standard methyl group [33].

**Scheme 10:**
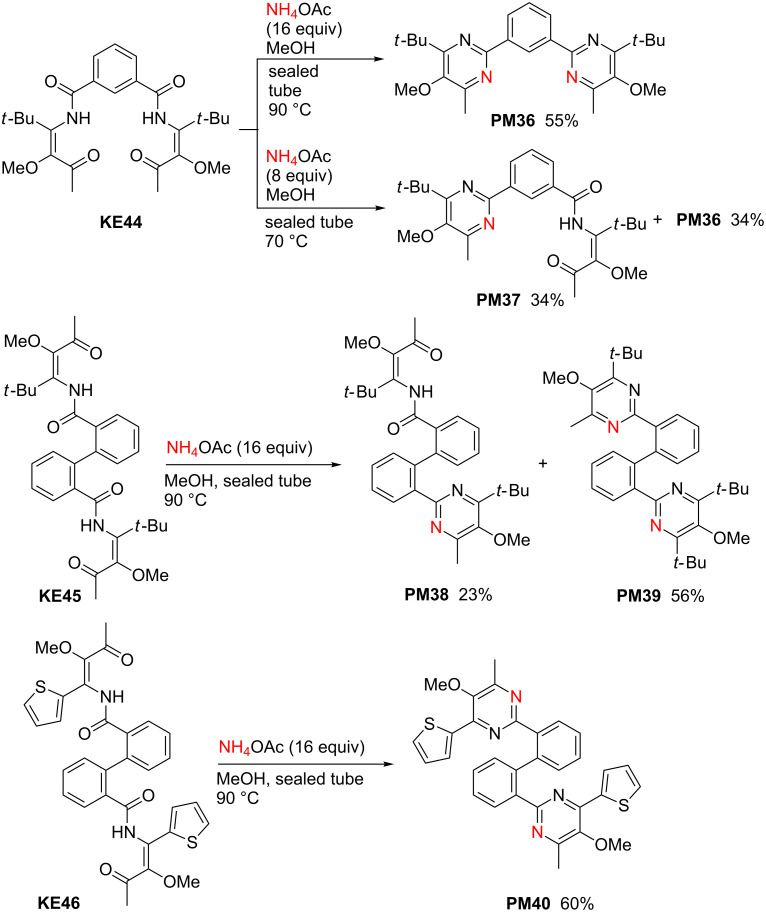
Synthesis of bis-pyrimidine derivatives **PM36**, **PM39** and **PM40** from β-ketoenamides **KE44**–**46** by method A (NH_4_OAc, MeOH, 60 °C).

The bis-β-ketoenamides **KE44**–**46** also provide the expected bis-pyrimidine derivatives (Scheme 10) [41]. With precursor **KE44** a full conversion into the expected bis-pyrimidine product **PM36** was achieved in 55% yield after 48 h reaction time, when a large excess of ammonium acetate (16 equiv) is employed, whereas with only eight equivalents (reaction time: 36 h) a 1:1 mixture of **PM36** and the intermediate mono-pyrimidine **PM37** – containing still one β-ketoenamide moiety – was isolated. The use of sixteen equivalents ammonium acetate afforded good yields of bis-pyrimidine derivative **PM39** and **PM40**, whereas in the case of **KE45** as starting material, considerable amounts of the mono-pyrimidine derivative **PM38** were isolated as side product. Subsequently, both mono-pyrimidine derivatives **PM37** and **PM38** were subjected to alternative cyclization reactions involving the remaining β-ketoenamide moiety [41]. It is worth mentioning that the relatively complex heterocyclic compounds depicted in Scheme 10 are accessible through the three-component reaction and subsequent condensation reaction in only two steps.

### Functionalization of pyrimidine derivatives

**Scheme 11:**
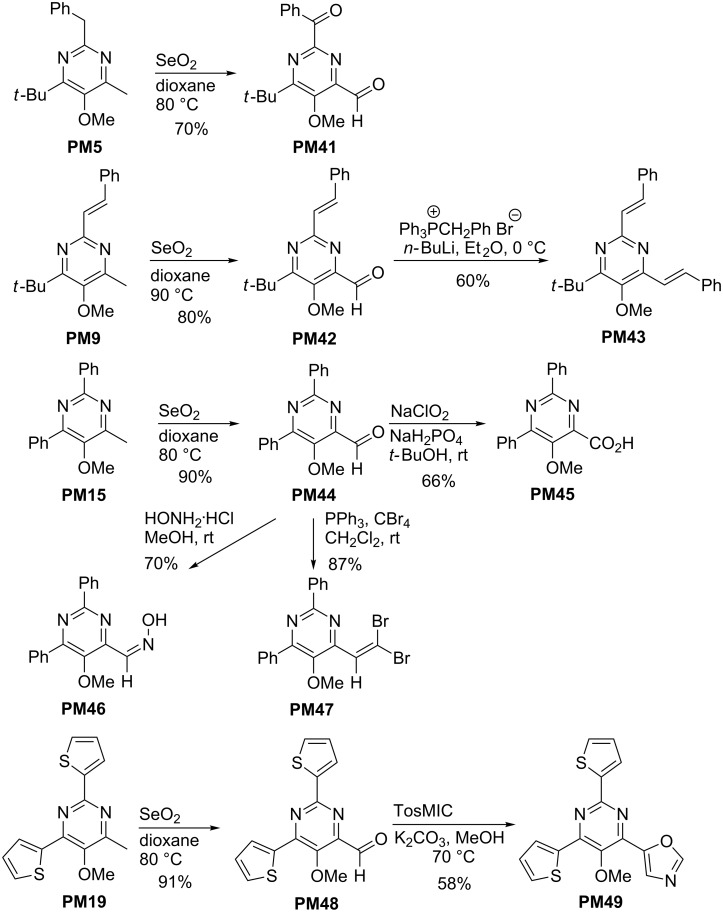
Functionalization of pyrimidine derivatives **PM** through selenium dioxide oxidations of **PM5**, **PM9**, **PM15** and **PM19** leading to 4-formyl-substituted pyrimidines **PM41**, **PM42**, **PM44** and **PM48** and selected subsequent transformations (TosMIC = tosylmethyl isocyanide).

The substitution pattern of the prepared pyrimidine derivatives **PM** allows a variety of subsequent transformations to new derivatives. The C-4 methyl (in one case benzyl) group is an inevitable structural feature of these pyrimidines, but it can smoothly be used for oxidation reactions to introduce new functional groups. As typical examples selenium dioxide oxidations of **PM5**, **PM9**, **PM15** and **PM19** furnishing aldehydes **PM41**, **PM42**, **PM44** and **PM48** are shown in Scheme 11 [33]. In case of the benzyl-substituted substrate **PM5**, the (probably faster) oxidation of the C-2 benzyl group could not be avoided and hence the dicarbonyl compound **PM41** was isolated [50]. The formyl group of the prepared intermediates allows further conversion into other functional groups as depicted in the scheme. Wittig reactions provided 4-alkenyl-substituted pyrimidine derivatives such as **PM43** or **PM47**, whereas further oxidation of **PM44** afforded the carboxylic acid **PM45** in good yield [33]. Alternatively, the conversion of **PM44** into oxime **PM46** or a van Leusen oxazole synthesis [51] of **PM48** with tosylmethyl isocyanide giving **PM49** were possible. The synthesis of pyrimidine derivative **PM49** with three heterocyclic substituents is remarkable and stresses the flexibility of the methods presented here.

**Scheme 12:**
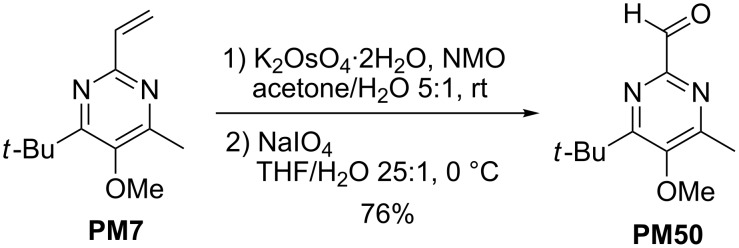
Conversion of 2-vinyl-substituted pyrimidine **PM7** into aldehyde **PM50**; (NMO = *N*-methylmorpholine *N*-oxide).

The easily introduced C-2 alkenyl groups may also be oxidized. Thus, dihydroxylation of the vinyl group in **PM7** followed by oxidative cleavage afforded pyrimidine derivative **PM50** having a formyl group at C-2 (Scheme 12) [31].

**Scheme 13:**
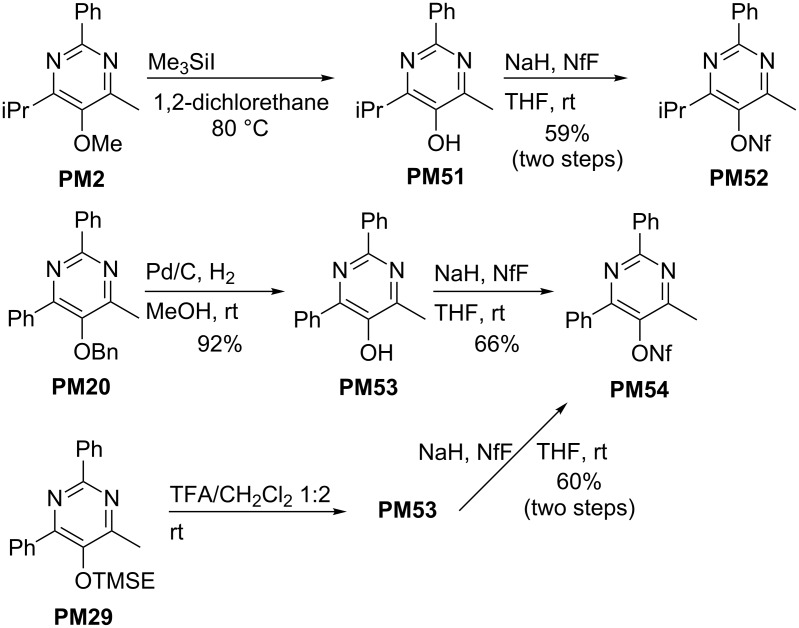
Deprotection of 5-alkoxy-substituted pyrimidines **PM2**, **PM20** and **PM29** and conversion into nonaflates **PM52** and **PM54**; (Nf =1-nonafluorobutanesulfonyl).

Next, the conversion of the 5-alkoxy groups of the pyrimidine derivatives **PM** into a 1-nonafluorobutanesulfonate group is presented, that – like the closely related triflate group – allows transition metal-catalyzed coupling reactions or nucleophilic substitutions [52]. The selected three examples presented in Scheme 13 show striking differences in the deprotection step [33]. The methoxy-substituted compound **PM2** requires harsh conditions employing trimethylsilyl iodide at 80 °C to provide the intermediate hydroxy derivative **PM51**. In contrast, the removal of the benzyl group in **PM20** can be achieved by palladium-catalyzed hydrogenolysis at room temperature to give hydroxy compound **PM53**. This method is certainly not applicable to pyrimidines with alkenyl substituents, but in this case 2-(trimethylsilyl)ethoxy-substituted compounds such as **PM29** can be used, whose deprotection with trifluoroacetic acid proceeds at room temperature. The obtained 5-hydroxy-pyrimidines can be purified and characterized or, for further transformations, the crude products are directly converted into the corresponding nonaflates by deprotonation with sodium hydride and treatment with 1-nonafluorobutanesulfonyl fluoride (NfF). Scheme 13 shows two examples, **PM52** and **PM54** that are ready for palladium-catalyzed reactions.

**Scheme 14:**
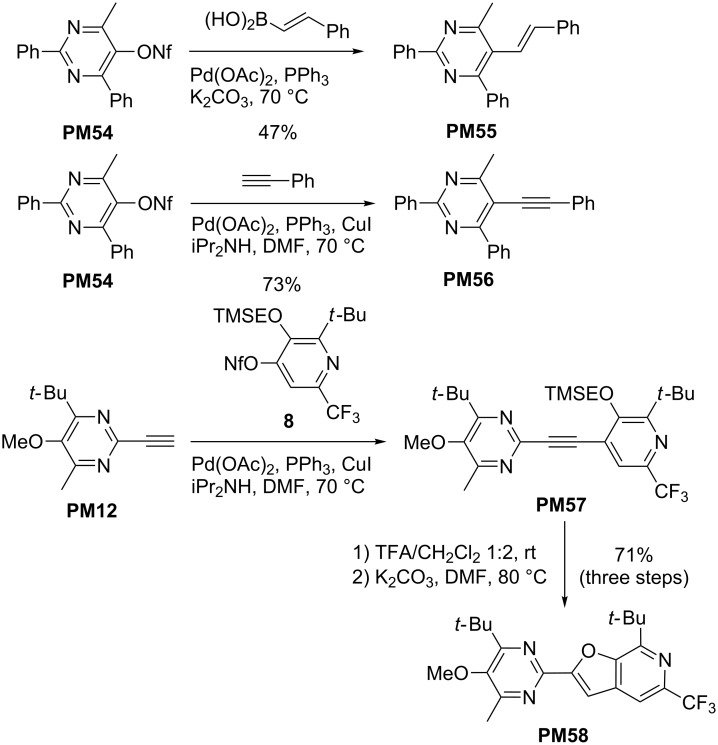
Palladium-catalyzed coupling reactions of **PM54** and **PM12** giving rise to new pyrimidine derivatives **PM55**–**58**.

As mentioned above, pyridyl nonaflates derived from the β-ketoenamides **KE** are excellent substrates for palladium-catalyzed coupling reactions as briefly discussed in our review [23]. Pyrimidyl nonaflates can analogously be used to achieve higher substitution degrees as illustrated by the examples shown in Scheme 14 [33]. Nonaflate **PM54** underwent a Suzuki–Miyaura reaction to **PM55** or a Sonogashira coupling to **PM56** under standard conditions. The ethynyl-substituted pyrimidine derivative **PM12** could also be employed in C–C coupling reactions as shown by its connection to pyridyl nonaflate **8** – readily available from β-ketoenamide **KE61** [33] – efficiently furnishing the disubstituted alkyne **PM57**. This intermediate was directly converted into pyrimidyl-substituted furopyridine derivative **PM58** in very good overall yield. The example of compound **PM58** nicely demonstrates the combination of different heterocycles that were generated from the two β-ketoenamides **KE20** and **KE61** and shows the potential of these methods in heterocyclic chemistry.

**Scheme 15:**
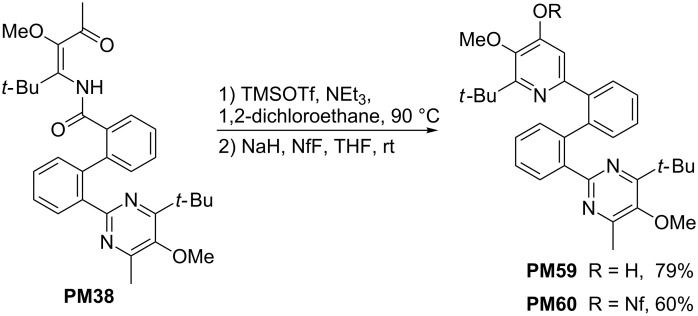
Synthesis of pyrimidyl-substituted pyridyl nonaflate **PM60**.

Options for palladium-catalyzed reactions are also offered by compound **PM60** that was prepared from mono-pyrimidyl-substituted β-ketoenamide **PM38** (see Scheme 10). This compound was converted into **PM59** by the standard cyclocondensation reaction (Scheme 15) leading to a pyridin-4-ol moiety that was converted to the nonaflate. Compound **PM60** bears a pyrimidyl and a pyridinyl substituent at the 2,2’-position of the biphenyl part [41].

### Synthesis of pyrimidine *N*-oxide derivatives

**Scheme 16:**
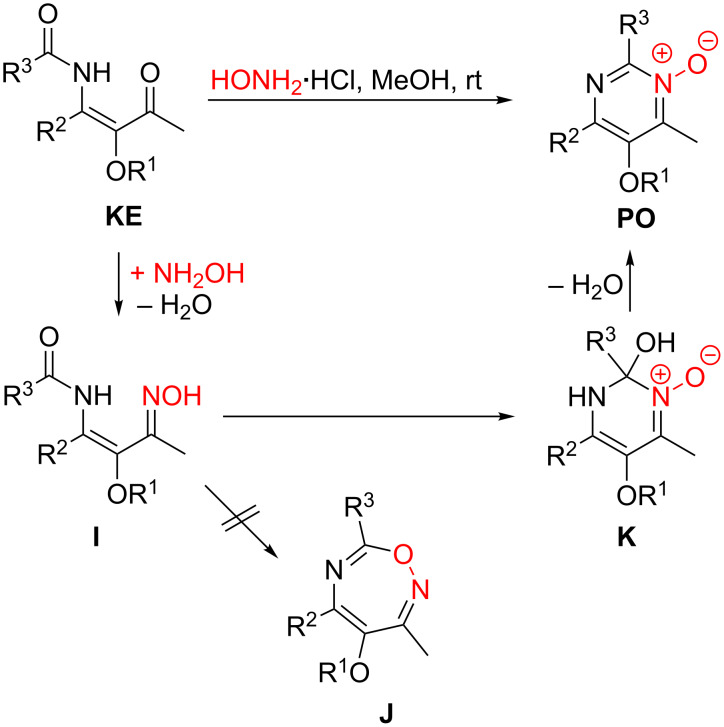
Condensation of LANCA-derived β-ketoenamides **KE** with hydroxylamine hydrochloride leading to pyrimidine *N*-oxides **PO**.

The condensation of β-ketoenamides **KE** with hydroxylamine hydrochloride can either deliver pyrimidine *N*-oxides **PO** or oxazepine derivatives **J**. However, only the six-membered heterocycles were isolated under the conditions employed (Scheme 16) [32]. Remarkably, the condensations occurred under milder conditions compared with those involving ammonium salts and smoothly provided the pyrimidine *N*-oxides at room temperature. An additional advantage of this approach to the pyrimidine skeleton is the fact that the *N*-oxide moiety could be exploited for the functionalization of the adjacent 4-alkyl group.

**Scheme 17:**
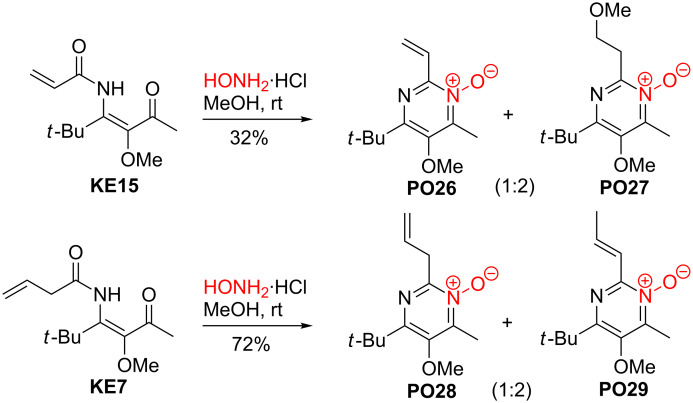
Reactions of β-ketoenamides **KE15** and **KE7** with hydroxylamine hydrochloride leading to pyrimidine *N*-oxides **PO26**–**29**.

**Scheme 18:**
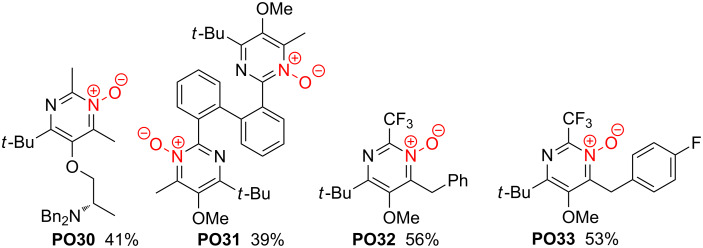
Structures of pyrimidine *N*-oxides **PO30**–**33** derived from β-ketoenamides **KE43**, **KE45**, **KE78** and **KE80**.

The scope of this method is again very broad as demonstrated by the 25 examples compiled in Table 5 and those of Scheme 17 and Scheme 18. This condensation method is compatible with all substituents that are available by the three-component reactions to β-ketoenamides **KE**, however, due to the slightly acidic reaction conditions the *tert*-butyldimethylsilyl protection group of **KE40** is removed during the formation of **PO7** (Table 5, entry 7).

**Table 5:** Preparation of pyrimidine *N*-oxides **PO1**–**25** through condensation of β-ketoenamides **KE** with hydroxylamine hydrochloride.^a^

entry	precursor	R^1^	R^2^	R^3^	product	yield	ref.

1	**KE3**	Me	iPr	CCl_3_	**PO1**	28%	[28]
2	**KE4**	Me	iPr	Ph	**PO2**	61%	[32]
3	**KE6**	Me	cPr	C_6_H_4_-4-Br	**PO3**	69%	[30]
4	**KE8**	Me	*t*-Bu	Bn	**PO4**	81%	[30]
5	**KE9**	Me	*t*-Bu	cPr	**PO5**	54%	[53]
6	**KE10**	Me	*t*-Bu	CH_2_CH_2_SiMe_3_	**PO6**	46%	[30]
7	**KE40**	Me	*t*-Bu	CH(OH)Me	**PO7**	30%	[43]
8	**KE14**	Me	*t*-Bu	CCl_3_	**PO8**	71%	[28]
9	**KE16**	Me	*t*-Bu	HC=CH-Me	**PO9**	54%	[54]
10	**KE17**	Me	*t*-Bu	HC=CH-Ph	**PO10**	88%	[32]
11	**KE18**	Me	*t*-Bu	HC=CH-2-Fu	**PO11**	99%	[32]
12	**KE19**	Me	*t*-Bu	HC=CH-2-Th	**PO12**	91%	[32]
13	**KE21**	Me	*t*-Bu	Ph	**PO13**	97%	[32]
14	**KE22**	Me	Ad	cPr	**PO14**	67%	[32]
15	**KE25**	Me	Ph	CCl_3_	**PO15**	96%	[28]
16	**KE27**	Me	Ph	HC=CH-Ph	**PO16**	58%	[54]
17	**KE29**	Me	Ph	Ph	**PO17**	58%	[30]
18	**KE34**	Me	2-Th	HC=CH-Ph	**PO18**	65%	[30]
19	**KE35**	Me	2-Th	2-Th	**PO19**	59%	[32]
20	**KE50**	Bn	*t*-Bu	cPr	**PO20**	38%	[30]
21	**KE58**	TMSE	cPr	cPr	**PO21**	65%	[32]
22	**KE59**	TMSE	cPr	HC=CH-Ph	**PO22**	45%	[30]
23	**KE63**	TMSE	*t*-Bu	HC=CH-Me	**PO23**	47%	[54]
24	**KE67**	TMSE	*t*-Bu	Ph	**PO24**	quant	[30]
25	**KE68**	TMSE	Ad	cPr	**PO25**	84%	[32]

^a^Abbreviations: Ad = 1-adamantyl, Fu = furyl, Py = pyridyl, Th = thienyl; all alkenyl substituents are *E*-configured.

Under the conditions applied the vinyl-substituted β-ketoenamide **KE15** furnished the expected pyrimidine *N*-oxide **PO26**. However, the addition of the solvent methanol to the double bond provided compound **PO27** as major product (Scheme 17) [32]. It was not studied whether the use of other solvents can suppress this addition reaction. The allyl-substituted β-ketoenamide **KE7** was converted under the standard conditions into condensation product **PO28**, but in this case a second compound, **PO29** bearing a shifted double bond, was isolated as main product [32].

A few pyrimidine *N*-oxides with special substituents are depicted in Scheme 18. They are generated from the enantiopure β-ketoenamide **KE43** [30], the biphenyl derivative **KE45** [41] and the aryl-substituted β-ketoenamides **KE78** and **KE80** [46]. In all cases, the corresponding heterocycles **PO30**–**33** were isolated in moderate to good yields.

### Typical subsequent reactions of pyrimidine *N*-oxides

**Scheme 19:**
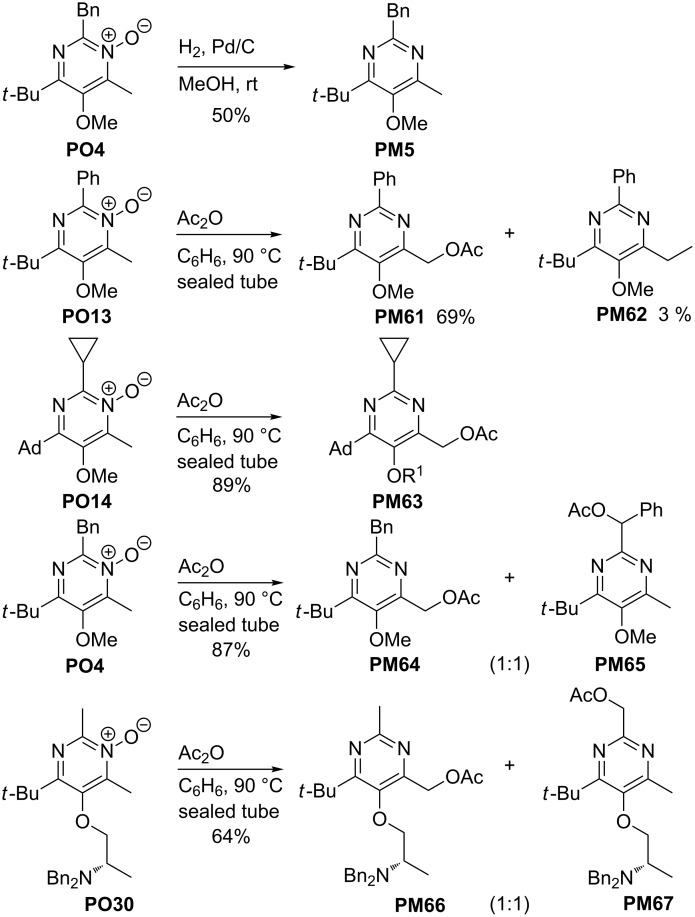
Reduction of **PO4** to **PM5** and Boekelheide rearrangements of **PO13**, **PO14**, **PO4** and **PO30** to 4-acetoxymethyl-substituted pyrimidine derivatives; Ad = 1-adamantyl.

The *N*-oxide moiety of pyrimidine *N*-oxides can easily be reduced by various methods, as shown by the reduction of **PO4** with hydrogen/palladium to give pyrimidine **PM5** (Scheme 19) [30]. Although compounds such as **PM5** are also directly available by condensation with ammonium salts (see above), the detour via pyrimidine *N*-oxides may have advantages in certain cases due to the milder reaction conditions of the condensation step. However, a more important transformation of pyrimidine *N*-oxides **PO** represents the Boekelheide rearrangement [55] to afford 4-acetoxymethyl-substituted pyrimidines and some typical examples of this side-chain functionalization are depicted in Scheme 19. Treatment of pyrimidine *N*-oxide **PO13** with acetic anhydride at 90 °C furnished the expected pyrimidine derivative **PM61** in 69% yield [32] showing that this transformation involves an internal redox reaction. However, the mechanism of this rearrangement is still under discussion [56–57] and side-products such as **PM62** having a 4-ethyl group (3% yield) and other compounds evidence the participation of radicals [30]. After the efficient conversion of pyrimidine *N*-oxide **PO14** into pyrimidine **PM63** no products of this type were isolated. The regioselectivity is another important feature of the Boekelheide rearrangement if alkyl groups are present at C-2 or C-4 next to the *N*-oxide moiety. The pyrimidine *N*-oxide **PO4** offers a benzyl substituent and a methyl group whereas **PO30** bears two methyl groups. In both cases, 1:1 mixtures of the two possible rearranged products, **PM64** and **PM65** [50] or **PM66** and **PM67** [30] were isolated, respectively.

**Scheme 20:**
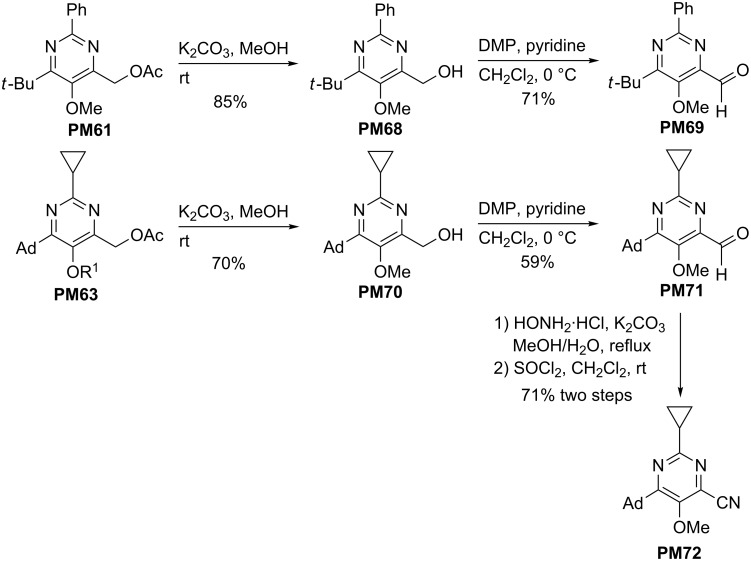
Deprotection of 4-acetoxymethyl-substituted pyrimidine derivatives **PM61** and **PM63**, oxidations to formyl-substituted pyrimidines **PM69** and **PM71** and synthesis of nitrile **PM72** (DMP = Dess–Martin periodinane).

4-Acetoxymethyl-substituted pyrimidine derivatives offer many options for the introduction of new substituents. For example the removal of the acetyl group by treatment with potassium carbonate in methanol and oxidation with Dess–Martin periodinane (DMP) converted **PM61** and **PM63** into aldehydes **PM69** and **PM71** in reasonable overall yields (Scheme 20) [30]. This pathway via the pyrimidine *N*-oxides represents a good alternative to the direct oxidation of the 4-methyl group by selenium dioxide (see Scheme 11). The subsequent transformation of the aldehyde **PM71** to the oxime followed by dehydration afforded nitrile **PM72** in good yield [30]. The latter should be a suitable precursor for three-component reactions with alkoxyallenes and carboxylic acids to furnish new β-ketoenamides **KE** bearing a 6-adamantyl-2-cyclopropyl-5-methoxypyrimidin-4-yl substituent. This again stresses the flexibility and versatility of our approach to complex heterocycles.

**Scheme 21:**
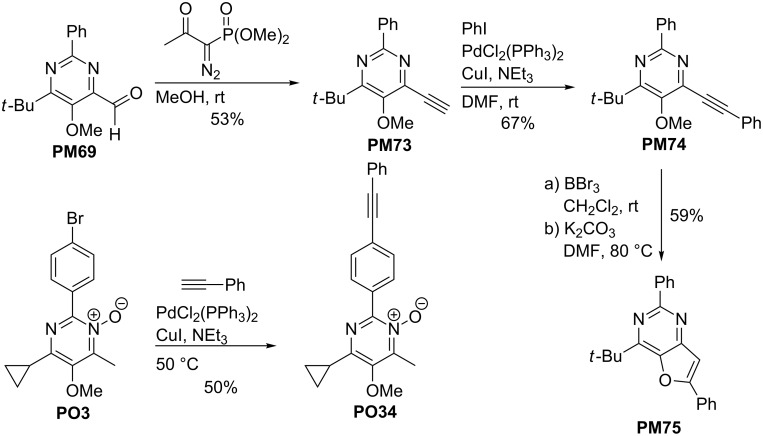
Synthesis of pyrimidinyl-substituted alkyne **PM74** and conversion into furopyrimidine **PM75** and Sonogashira reaction of **PO3** with ethynylbenzene to pyrimidine *N*-oxide **PO34**.

The aldehyde **PM69** was further converted into the terminal alkyne **PM73** by employing the Bestmann–Ohira protocol (Scheme 21). After its Sonogashira reaction with iodobenzene to the intermediate disubstituted alkyne **PM74** this compound was converted into furopyrimidine derivative **PM75** [30]. Finally, the bromoaryl group in **PO3** was engaged in a coupling with ethynylbenzene to give **PO34**. This latter reaction proves that the *N*-oxide moiety is compatible with palladium/copper-catalyzed reactions [30]. The conversion of the 5-alkoxy substituent into a nonafloxy group (as shown above with the pyrimidine derivatives, see Scheme 13) was not examined so far, however, it should be possible. Hence, the pyrimidine *N*-oxides may also be used in other palladium-catalyzed processes in order to introduce new substituents at C-5 of the heterocycles.

### Synthesis of oxazole derivatives

**Scheme 22:**
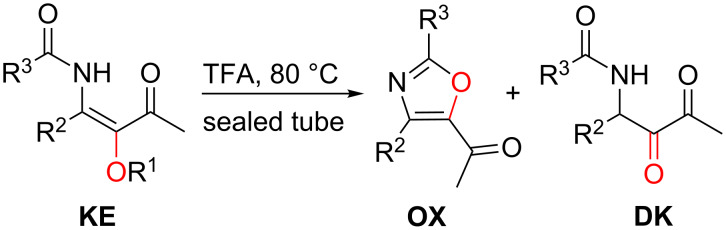
Trifluoroacetic acid-promoted conversion of LANCA-derived β-ketoenamides **KE** into oxazoles **OX** and 1,2-diketones **DK**.

By brief heating with trifluoroacetic acid β-ketoenamides **KE** with acid-labile alkoxy substituents OR^1^ underwent an unexpected formation of 5-acetyl-substituted oxazole derivatives **OX** (Scheme 22) [42,45]. This useful transformation proceeds with benzyloxy-, *p*-methoxybenzyl-, 2-tetrahydropyranyl- and 2-(trimethylsilyl)ethoxy-substituted β-ketoenamides **KE** as precursors and - mainly depending on the size of substituent R^2^ - oxazoles **OX** and/or the simple hydrolysis products 1,2-diketones **DK** were isolated in moderate to excellent yields (Table 6). With substituents R^2^ of moderate bulkiness the oxazoles **OX** are formed exclusively (Table 6, entries 1, 3–6, 17, and 20–23), whereas for the two compounds **KE57** and **KE58** (R^2^ = R^3^ = cyclopropyl) the corresponding oxazoles **OX6** and **OX7** were isolated as highly predominating products (Table 6, entries 7 and 8), but traces of the corresponding 1,2-diketones **DK2** and **DK3** were detected in the crude product. The reactions of β-ketoenamides **KE60**, **KE61** and **KE68** (R^2^ = *tert*-butyl or adamantanyl, R^3^ = methyl, trifluoromethyl or cyclopropyl) provided mixtures of oxazoles **OX7**, **OX8** and **OX10**, respectively, and of 1,2-diketones **DK4**, **DK5** and **DK11** (Table 6, entries 9, 10, and 16). For examples with very bulky substituents R^2^ and R^3^ the exclusive formation of the 1,2-diketones **DK1** and **DK6**–**10** was observed (Table 6, entries 2, and 11–15). Trifluoroacetic acid treatment of β-ketoenamides **KE70** and **KE71** not only furnished **DK12** and **DK13** in moderate yield, but also dimeric products whose structure has still to be established [43].

**Table 6:** Preparation of oxazoles **OX1**–**15** and 1,2-diketones **DK1**–**13** through trifluoroacetic acid-promoted reaction of β-ketoenamides **KE**.^a^

entry	**KE**	R^1^	R^2^	R^3^	**OX**	yield	**DK**	yield	ref.

1	**KE48**	Bn	*n*-Non	Ph	**OX1**	51%	–		[45]
2	**KE51**	Bn	*t-*Bu	2-Th	–	–	**DK1**	83%	[45]
3	**KE52**	Bn	Ph	CF_3_	**OX2**	74%	–	–	[45]
4	**KE53**	Bn	Ph	Ph	**OX3**	48%	–	–	[45]
5	**KE54**	Bn	Ph	2-Py	**OX4**	64%	–	–	[45]
6	**KE56**	PMB	Ph	CF_3_	**OX5**	53%	–	–	[45]
7	**KE57**	2-THP	cPr	cPr	**OX6**	51%	**DK2**	<1%	[45]
8	**KE58**	TMSE	cPr	cPr	**OX7**	67%	**DK3**	<1%	[45]
9	**KE60**	TMSE	*t-*Bu	Me	**OX8**	24%	**DK4**	29%	[45]
10	**KE61**	TMSE	*t-*Bu	CF_3_	**OX9**	61%	**DK5**	31%	[45]
11	**KE62**	TMSE	*t-*Bu	CH=CH_2_	–	–	**DK6**	73%	[43]
12	**KE63**	TMSE	*t-*Bu	CH=CH-Me	–	–	**DK7**	80%	[43]
13	**KE64**	TMSE	*t-*Bu	CH=CH-Ph	–	–	**DK8**	60%	[43]
14	**KE65**	TMSE	*t-*Bu	CH=CH-C_6_H_4_-NO_2_	–	–	**DK9**	55%	[43]
15	**KE66**	TMSE	*t-*Bu	CH=CH-2-Fu	–	–	**DK10**	70%	[43]
16	**KE68**	TMSE	Ad	cPr	**OX10**	12%	**DK11**	65%	[45]
17	**KE69**	TMSE	Ph	CF_3_	**OX11**	98%	--	--	[45]
18	**KE70**	TMSE	Ph	CH=CH-Me	–	–	**DK12**	30%^b^	[43]
19	**KE71**	TMSE	Ph	CH=CH-Ph	–	–	**DK13**	33%^b^	[43]
20	**KE72**	TMSE	Ph	C≡CH	**OX12**	57%	–	–	[45]
21	**KE74**	TMSE	Ph	2-Py	**OX13**	99%	–	–	[45]
22	**KE75**	TMSE	Ph	2-Th	**OX14**	68%	–	–	[45]
23	**KE76**	TMSE	Ph	Ac	**OX15**	39%	–	–	[45]

^a^Abbreviations: Ad = 1-adamantyl, Py = pyridyl, Fu = furyl, Th = thienyl, Ac = acetyl; all alkenyl substituents are *E*-configured. ^b^In addition, ca. 30% of a dimeric compound were isolated.

**Scheme 23:**
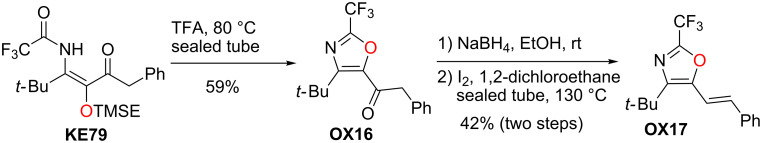
Conversion of β-ketoenamide **KE79** into oxazole **OX16** and transformation into 5-styryl-substituted oxazole **OX17**.

The method could also be extended to aryl-substituted β-ketoenamide **KE79** that delivered oxazole derivative **OX16** in 59% yield (Scheme 23) [45]. A subsequent reduction of the carbonyl group followed by iodine-induced elimination gave the 5-styryl-substituted oxazole **OX17**. This sequence demonstrates the potential of the C-5 functionalized oxazoles to be used for further transformations (see below).

**Scheme 24:**
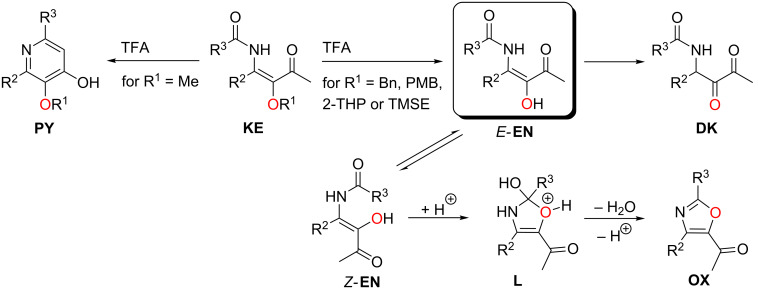
Mechanisms of the formation of 1,2-diketones **DK** and of acetyl-substituted oxazole derivatives **OX**.

The examples collected in Table 6 show a dichotomy of oxazole and 1,2-diketone formation that is not fully understood so far. As mentioned above, the presence of bulky substituents R^2^ (and R^3^) seems to be a prerequisite of the 1,2-diketone formation, however, for the series with R^2^ = phenyl the observed product distributions are not easy to explain (Table 6, entries 18–23). Nevertheless, a plausible mechanism is presented in Scheme 24 showing the analogy to the Gabriel–Robinson oxazole synthesis [58]. For β-ketoenamides **KE** with OR^1^ groups that are not easily cleaved by acids the cyclization to pyridin-4-ol derivatives **PY** occurs without touching of the alkoxy group. If this group is reacting with trifluoroacetic acid the *E*-configured enol *E-***EN** is generated first and its prototropy directly delivers the isolated 1,2-diketones **DK**. Experiments with labelled oxygen showed that the oxazole oxygen originates from the alkoxy group and not from the amide moiety [45]. The oxazole formation therefore requires a configurational switch from enol *E-***EN** to *Z-***EN**. Very likely, this step is acid-catalyzed as the subsequent cyclization to form the five-membered intermediate **L** and the final water elimination to oxazole **OX**. The formation of *Z*-**EN** is possibly disfavored by bulky groups R^2^ due to repulsion with the acetyl group. The cyclization step leading to **L** may also be hampered if R^3^ is too bulky. In these cases, no sufficient concentrations of *Z*-**EN** or of **L** are formed and hence the 1,2-diketones **DK** are obtained as the products. It should also be mentioned that isolated 1,2-diketones **DK** do not undergo cyclizations to **OX** even after extended treatment with trifluoroacetic acid.

**Scheme 25:**
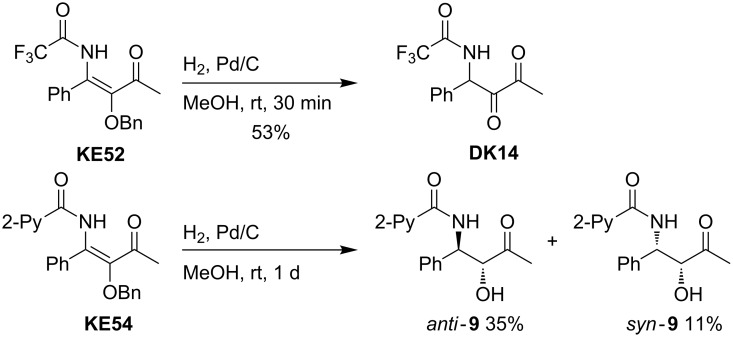
Hydrogenolyses of benzyloxy-substituted β-ketoenamides **KE52** and **KE54** to 1,2-diketone **DK14** and to diastereomeric α-hydroxy-β-amino ketones **9**.

As an alternative to the strongly acidic conditions, palladium-catalyzed hydrogenolysis of the benzyloxy-substituted derivatives is possible, thus avoiding the condensation to oxazoles. Scheme 25 shows the conversion of **KE52** into 1,2-diketone **DK14** (compare entry 3 of Table 6). Longer reaction times lead to a subsequent reduction of the internal carbonyl group as shown by the conversion of **KE54** into the two diastereomeric α-hydroxy-β-amino ketones **9** [45]. Due to the moderate mass balance of this transformation we cannot exclude that the second carbonyl group was also partially reduced.

### Subsequent reactions of oxazole derivatives

**Scheme 26:**
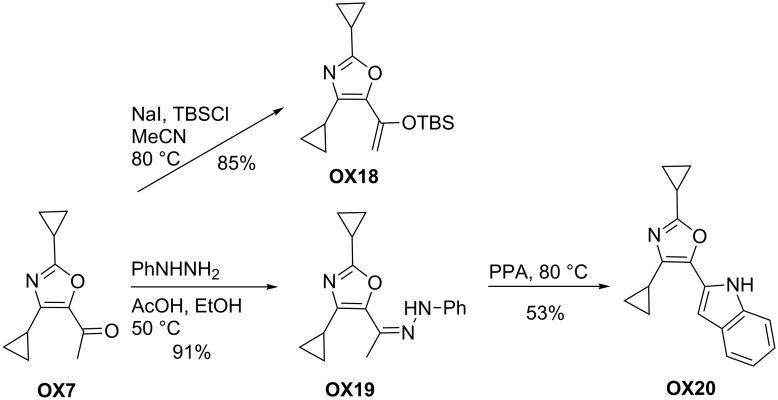
Conversions of 2,4-dicyclopropyl-substituted oxazole **OX7** into oxazole derivatives **OX18**–**20** (PPA = polyphosphoric acid).

**Scheme 27:**
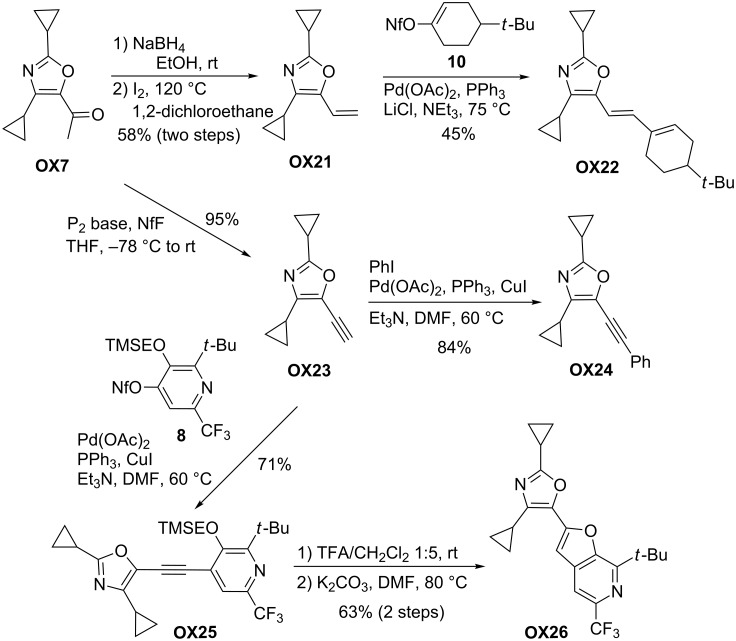
Syntheses of vinyl and ethynyl-substituted oxazole derivatives **OX21** and **OX23** and their palladium-catalyzed reactions to **OX22** and **OX24**–**26** (Schwesinger P_2_ base).

As already shown in Scheme 23, the carbonyl group at C-5 of oxazole derivatives **OX** offers possibilities for subsequent reactions to other functionalized oxazoles. Typical examples are depicted in Scheme 26 and Scheme 27 employing 2,4-dicyclopropyl-substituted oxazole **OX7** as the starting material. The efficient conversion of the acetyl group into the corresponding silyl enol ether moiety delivered **OX18** that may be used for further transformations. Alternatively, **OX7** and phenyl hydrazine afforded the corresponding hydrazone **OX19** in excellent yield that was further treated with polyphosphoric acid to undergo a Fischer indole reaction to 5-indolyl-substituted oxazole **OX20**.

**Scheme 28:**
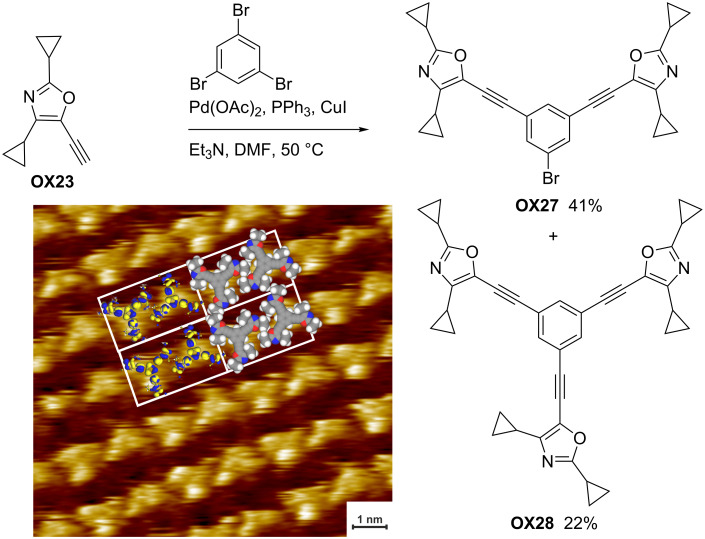
Synthesis of *C*_3_-symmetric oxazole derivative **OX28** and the STM current image of its 1-phenyloctane solution on highly oriented pyrolytic graphite (HOPG).

To demonstrate the versatility of the route to new oxazole derivatives, typical palladium-catalyzed processes are compiled in Scheme 27. First, the acetyl moiety was converted into a vinyl or an ethynyl substituent. The reduction of **OX7** followed by elimination to **OX21** proceeded smoothly and as subsequent transformation a Heck reaction with alkenyl nonaflate **10** was performed delivering **OX22**. The conversion of **OX7** to alkyne **OX23** applied the protocol of Lyapkalo et al. [59] using Schwesinger’s base [60] as crucial reagent. First, the corresponding nonaflate is generated from **OX7** that immediately underwent elimination to the alkyne. Ethynyl-substituted oxazole **OX23** was isolated in excellent yield and subsequently employed in Sonogashira couplings. Iodobenzene afforded compound **OX24** in high yield and (β-ketoenamide-based) pyridinyl nonaflate **8** gave **OX25**. The removal of the TMSE group by acid treatment and subsequent cyclization furnished the furopyridyl-substituted oxazole derivative **OX26** in good overall yield [45]. The examples shown in Scheme 27 and Scheme 28 demonstrate the manifold options to synthesize complex heterocyclic systems by the building block system derived from β-ketoenamides **KE**.

Finally, the synthesis of star-shaped compound **OX28** is presented. A threefold Sonogashira reaction of 1,3,5-tribromobenzene with ethynyl-substituted **OX23** gave the desired **OX28** in 22% yield; the major product (41%) of this experiment was the double-coupling product **OX27** and the mono-coupling product (not shown) was isolated in 5% yield [45]. A solution of the *C*_3_-symmetric compound **OX28** in 1-phenyloctane was investigated by scanning tunneling microscopy (STM) to reveal its ability to form self-assembled monolayers at the interface with highly oriented pyrolytic graphite (HOPG). The STM current image inserted in Scheme 28 shows bright areas that indicate the positions of the π-systems, whereas the dark areas indicate the cyclopropyl groups.

### Synthesis of quinoxalines

**Scheme 29:**
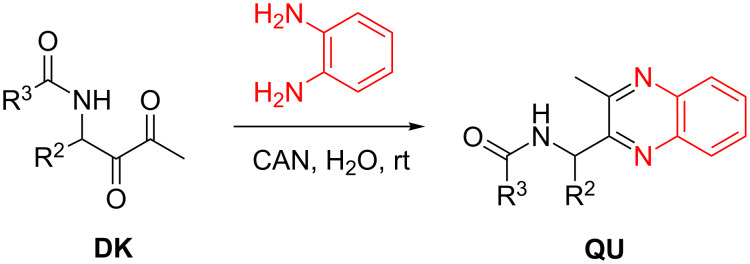
Condensation of 1,2-diketones **DK** with *o*-phenylenediamine to quinoxalines **QU1**–**7** (CAN = cerium ammonium nitrate).

The acylamido-substituted 1,2-diketones **DK** obtained by hydrolysis of several β-ketoenamides **KE** also offer possibilities of further synthetic applications. The reduction to α-hydroxy-β-amino ketones such as compound **9** has been already mentioned (see Scheme 25), but the vicinal carbonyl groups may also be employed for condensation reactions leading to heterocycles, for instance the Radziszewski reaction to imidazoles [61–62]. As an example, the condensation of 1,2-diketones **DK** with *o*-phenylenediamine to quinoxalines **QU** [63] employing cerium ammonium nitrate [64] as catalyst was investigated (Scheme 29). This transformation proceeded smoothly at room temperature in water as solvent and provided the expected acylamido-substituted quinoxalines **QU1**–**7** in moderate to good yields (Table 7) [43,45].

**Table 7:** Preparation of quinoxalines **QU1**–**7** by condensation of 1,2-diketones DK with *o*-phenylenediamine.^a^

entry	**DK**	R^2^	R^3^	**QU**	yield	ref.

1	**DK1**	*t-*Bu	2-Th	**QU1**	41%	[45]
2	**DK6**	*t-*Bu	CH=CH_2_	**QU2**	51%	[43]
3	**DK7**	*t-*Bu	CH=CH-Me	**QU3**	58%	[43]
4	**DK8**	*t-*Bu	CH=CH-Ph	**QU4**	53%	[43]
5	**DK10**	*t-*Bu	CH=CH-2-Fu	**QU5**	55%	[43]
6	**DK11**	Ad	cPr	**QU6**	30%	[45]
7	**DK13**	Ph	CH=CH-Ph	**QU7**	42%	[43]

^a^Abbreviation: Ad = 1-adamantyl, Fu = furyl, Th = thienyl; all alkenyl substituents are *E*-configured.

## Conclusion

**Scheme 30:**
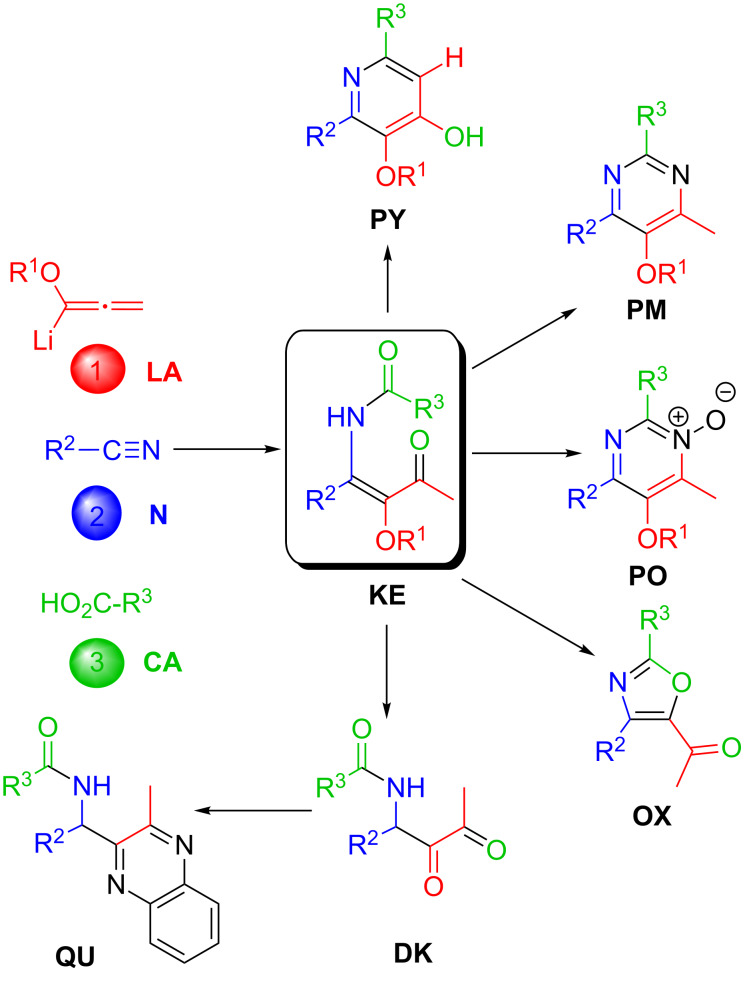
The LANCA three-component reaction leading to β-ketoenamides **KE** and the structure of functionalized pyridines **PY**, pyrimidines **PM**, pyrimidine *N*-oxides **PO**, oxazoles **OX**, 1,2-diketones **DK** and quinoxalines **QU** derived thereof.

Lithiated alkoxyallenes **LA**, nitriles **N** and carboxylic acids **CA** undergo a three-component reaction (LANCA reaction) that affords β-ketoenamides **KE** in good to very good yields (Scheme 30). The reaction proceeds through a unique mechanism being driven by the high energy level of the allenes. The eighty examples of β-ketoenamides **KE** collected in this review impressively demonstrate the broad scope of this three-component reaction that is compatible with all kinds of substituents R^2^ and R^3^ and several functional groups within these substituents. Enantiopure components efficiently lead to products with stereogenic centers. Dinitriles or dicarboxylic acids provide the expected bis-β-ketoenamides in moderate yield.

The prepared β-ketoenamides **KE** are excellent precursors for the synthesis of specifically substituted heterocycles (Scheme 30). The intramolecular aldol-type condensations leading to a manifold of pyridine derivatives **PY** was already subject of a review article [23]. In this report, we demonstrate that the β-ketoenamides **KE** are also excellent precursors for the synthesis of a variety of pyrimidines **PM**, pyrimidine *N*-oxides **PO**, 4-acetyl-substituted oxazoles **OX** and – via 1,2-diketones **DK** – of quinoxalines **QU**. The substitution pattern of all compounds allows specific subsequent reactions, for instance, by substitution of the alkoxy groups with a nonafloxy group all kinds of palladium-catalyzed coupling reactions. Specific oxidation reactions also lead to a variety of new heterocyclic compounds. All the examples collected here show the potential of this approach to highly functionalized heterocycles, furnishing compounds with a very high degree of structural diversity that should be of interest in drug synthesis or material science. The versatility of alkoxyallenes [11–20,65–66] as easily available C_3_ building blocks is key for this prosperousness.
